# Public health authorities in Germany during the SARS-CoV-2 pandemic – a constructive-critical reflection

**DOI:** 10.3205/dgkh000629

**Published:** 2026-02-24

**Authors:** Ursel Heudorf, René Gottschalk

**Affiliations:** 1 Former Deputy Head of the Frankfurt am Main Health Authority, Frankfurt am Main, Germany; 2 Former Head of the Frankfurt am Main Health Authority, Institute for Medical Virology, Goethe University, Frankfurt am Main, Germany

**Keywords:** SARS-CoV-2, COVID-19, pandemic, health authorities, public health service, reappraisal

## Abstract

**Introduction:**

Following the COVID-19 pandemic, a retrospective evaluation of the measures is still pending in Germany. This article will attempt to draw constructive and critical lessons from this pandemic for the actions of the public health authorities (PHA) in similar situations in the future.

**Results:**

First, the legal and professional basis of the public health authorities in Germany to deal with a pandemic is presented. However, the analysis of the work of the PHA during the pandemic shows that the health authorities imposed extensive restrictions on freedom due to political and administrative law requirements without implementing the pandemic plan, the technical evidence and the principles of administrative law. The lessons of history were also ignored.

**Discussion:**

The non-compliance with professional und legal requirements may be explained by mass phenomena that occurred during the pandemic. Simplified messages and narratives led to uncertainty and fear, blurring individual boundaries in favor of collective reactions. Within a very short time in spring 2020, a collective threat scenario had gripped the whole of society – including the public health services. Combating the virus became the only goal, which supposedly unconditionally required maximum measures and restrictions on freedom and did not sufficiently take into account the considerable consequences for society as a whole.

The question arises as to how, even in times of a pandemic, evidence and experience can continue to guide decisions instead of threat scenarios, as was possible in Sweden. There, the Swedish pandemic plan was implemented in accordance with the principle of proportionality.

**Conclusion:**

With our contribution to the discussion, we would like to encourage the PHA to take up this discussion and reappraisal – in responsibility for itself, for society and, in particular, for the people whose freedoms have been significantly restricted.

## Zusammenfassung

**Einleitung:**

Nach der SARS-CoV-2-Pandemie stehen die rückblickende Bewertung und Aufarbeitung der Maßnahmen in Deutschland weiterhin aus. In dem Beitrag soll versucht werden, konstruktiv-kritische Lehren aus dieser Pandemie für das Vorgehen des öffentlichen Gesundheitsdienstes (ÖGD) in künftigen vergleichbaren Situationen anzustoßen.

**Ergebisse:**

Zunächst werden die rechtlichen und fachlichen Grundlagen der Gesundheitsämter in Deutschland für den Umgang mit einer Pandemie dargelegt. Die Betrachtung der Arbeit des ÖGD während der Pandemie zeigt jedoch, dass die Gesundheitsämter aufgrund politischer und verwaltungsrechtlicher Vorgaben in großem Umfang Freiheitseinschränkungen verfügt haben, ohne den Pandemieplan, die fachliche Evidenz und die Grundlagen des Verwaltungsrechts umzusetzen. Auch blieben die Lehren aus der Geschichte unbeachtet.

**Diskussion:**

Erklärung für die Nicht-Beachtung der fachlichen und rechtlichen Grundlagen können die Massenphänomene bieten, die in der Pandemie auftraten. Vereinfachte Botschaften und Narrative führten zu Unsicherheit und Angst, wodurch individuelle Grenzen zugunsten kollektiver Reaktionen verschwammen. Innerhalb kürzester Zeit hatte im Frühjahr 2020 ein kollektives Bedrohungsszenario die gesamte Gesellschaft erfasst – einschließlich des ÖGD. Die Bekämpfung des Virus wurde einziges Ziel, das (vermeintlich) bedingungslos maximale Maßnahmen und Freiheitseinschränkungen erforderte und die erheblichen gesamtgesellschaftlichen Konsequenzen ungenügend berücksichtigte.

Es stellt sich die Frage, wie auch in Pandemiezeiten erreicht werden kann, dass Evidenz und Erfahrung statt Bedrohungsszenarien entscheidungsleitend bleiben können, wie es in Schweden möglich war, da deren Pandemieplan unter Beachtung des Verhältnismäßigkeitsprinzips umgesetzt wurde.

**Schlussfolgerung:**

Mit unserem Diskussionsbeitrag möchten wir anregen, dass der ÖGD diese Diskussion und Aufarbeitung aufnimmt – in Verantwortung für sich selbst, für die Gesellschaft, insbesondere aber die Menschen, deren Freiheiten erheblich eingeschränkt wurden.

## Introduction

The SARS-CoV-2 pandemic started five years ago and was declared to be over by the World Health Organization (WHO) two years ago. Some countries have started a comprehensive evaluation of their pandemic management, e.g., the UK and Sweden.

In the UK, for example, a COVID-19 inquiry is addressing various aspects of the SARS-CoV-2 pandemic in different modules. The “Module 1 Report – The resilience and preparedness of the United Kingdom” was published in 2024 [1] and the government subsequently responded to implement its recommendations [2]. Other modules, such as UK decision-making and political governance, impact of the Covid-19 pandemic on healthcare systems, vaccines, therapeutics, procurement, care sector, test strategies, contact tracing and isolation, children and young people, economic response, and impact on society are currently being worked on.

In Sweden, the Coronavirus Commission acknowledged that their strategy for economic crisis management was correct. The choice of path in terms of disease prevention and control focusing on advice and recommendations was also rated as good. “It meant that citizens retained more of their personal freedom than in many other countries.” However, there was criticism that measures should have been taken more quickly at the beginning of the pandemic and that older people and other at-risk groups should have been better protected [3].

In Germany, a committee of experts appointed by the federal government in 2021 published an initial analysis in 2022 of the measures taken in Germany in order to learn from them for future pandemics. However, in their report, the experts pointed out significant limitations to their work, including the fact that their assessment of the measures based on the Infection Protection Act (IfSG) was only requested after the event and that there was a lack of sufficient and rigorous accompanying data collection as a basis for evaluating individual measures or bundles of measures. The evaluation commission also pointed out fundamental deficits and areas for improvement in the data basis and data management, which, however, could not be comprehensively analyzed and evaluated due to time and resource constraints and partly because the appointed evaluation commission had only limited expertise in the disciplines of statistics and epidemiology [4].

In the meantime, investigation committees (Untersuchungsausschüsse) and commissions of inquiry (Enquete-Kommissionen) have been set up in some federal states in Germany. A national commission of inquiry started its work in autumn 2025. Thus, the much-demanded retrospective evaluation and review of the measures taken [5], [6] is still pending in Germany.

During the pandemic, public health authorities (PHAs) played a central role in implementing the politically mandated pandemic measures in Germany. In their corporate mission statement, German PHAs strive to increasingly meet modern civil society requirements and social challenges in addition to their regulatory duties. They are committed to scientific standards and evidence-based practices in their work [7]. 

With our contribution to the discussion, we would like to encourage the PHAs in Germany to take up this discussion and evaluation on their own initiative – against the backdrop of their legal, professional, and historical preconditions. 

We emphasize that the following is not an evaluation of how effective the measures taken (as individual measures or as a bundle) have been. For this, we refer to other articles and reviews, including Cochrane Reviews [8], [9], [10], [11], [12].

With a focus on the applied non-pharmaceutical pandemic measures, the questions to be answered are: Did the PHAs in Germany meet their own standards during the pandemic? Did they act in a professionally, science and evidence based manner and/or generated the necessary evidence for their actions? Did they observe the legal principle of proportionality and finally: have they learned from their history? 

## Background: structural, legal, and professional principles of federal public health authorities for infection control and pandemic management in Germany

The Federal Republic of Germany consists of 16 federal states. The healthcare system comprises inpatient and outpatient treatment of individuals, provided in non-profit or privately run hospitals and outpatient medical practices. In addition, the public healthcare system takes a preventive approach to population medicine, namely maintaining the health of the population. PHAs act on each of the three political levels – national, federal state, and local. All of them are fundamentally subordinate to the political level. The so-called higher national authorities, such as the Robert Koch Institute (RKI), are subordinate to the National Ministry of Health, federal health authorities to the federal-state health ministers, and basic health authorities to the corresponding local politicians. 

The tasks of the approximately 400 PHAs are laid down in the public health service laws of the federal states [13]. These include regulatory duties such as infection control with the handling of notifiable communicable diseases, infection control monitoring of medical care and community facilities such as schools, kindergartens, and refugee accommodations, environmental health protection, the screening of school-age children, occupational health measures, etc. In addition, their other duties include health advice, information, easily accessible services for specific population groups (e.g., social psychiatry and addiction prevention), but also health reporting and policy advice.

In addition to the above-mentioned federal state health service laws [13], the Infection Protection Act (IfSG) [14], a national law, is an essential legal basis for infection control in Germany. It applies in all federal states, is implemented in these states through legal regulations, and must be enforced by local health authorities.

When performing their regulatory duties, health authorities are bound by the constitution (Grundgesetz) of the Federal Republic of Germany as well as the principles of the rule of law. Before imposing measures that restrict freedom, it must always be examined whether there is a legitimate purpose and whether the means are appropriate to the intended purpose: the measure must be suitable, necessary, and appropriate. A legitimate purpose exists if the measure is aimed at the welfare of the general public. A measure is suitable if it achieves, or at least promotes, the intended purpose. It is necessary if there is no equally effective, less severe means of achieving the goal. It is appropriate if the relationship between the purpose and the means is proportionate. According to the prohibition of excessiveness, the “mildest measure” must be chosen to achieve the objective. In accordance with dutiful discretion (*pflichtgemäßes Ermessen*), the measures must also be adapted to the severity of the disease to be prevented, i.e., the more severe the disease, the more comprehensive the measures may be.

The first German National Pandemic Plan was published in 2005 based on the World Health Organization (WHO) pandemic plan and was revised in 2017 after plan exercises, staff framework exercises, and experience with the 2009 influenza pandemic [15], [16], [17]. Experience with the 2009 influenza pandemic [18] showed that the system with the phases containment, protection and mitigation, and recovery had proven its worth [19]. The “paternalistic communication” experienced at that time was recognized as a flaw, as it led to 1), a false sense of security or even uncertainty due to contradictory information and 2), anger and conspiracy theories if the information policy subsequently proved to be inappropriate [20]. 

With a view to future pandemics, rethinking was called for, including an “information policy that creates the conditions for enlightened and informed citizens” [20]. Among other things, suitable hospital surveillance or sentinel systems and recording systems for mortality, severe clinical courses, and the utilization of care structures were called for and implemented by the Robert Koch Institute (RKI) in the following years. 

The pandemic plan, which was originally focused on influenza, was updated in March 2020 by the RKI to adapt it to COVID-19 [21]. The defined goals and phases were basically retained, and the measures for hygiene and contact reduction, i.e., the non-pharmaceutical measures, were adopted. With regard to surveillance, reference was made to the possibilities and limitations of mandatory reporting data and the other surveillance systems that have since been established: ARE and Grippeweb for acute respiratory diseases and influenza-like illnesses [https://grippeweb.rki.de], SARI for severe acute respiratory diseases [sari.eu-burden.info], but also DIVI intensive care register (German Interdisciplinary Association for Intensive Care and Emergency Medicine) [https://www.intensivregister.de], the Integrated Care Record IVENA (interdisciplinary care record) [https://www.ivena.de] established in Hessen and other federal states, and EUROMOMO (European mortality monitoring) [https://www.euromomo.de] (see Table 1 (Tab. 1)).

## Public health authorities in Germany during the SARS-CoV-2 pandemic

### Evidence and evidence generation

Just a few weeks after the start of the pandemic, politicians deviated from the pandemic plan and enacted laws and regulations that extensively infringed on people’s fundamental rights. Containment measures were maintained, and the transition to the second phase of the pandemic plan, protection, was not initiated. Established surveillance instruments were not used. The primacy of preventing all infections, including asymptomatic ones, took priority over all other aspects of public welfare. 

The PHAs continued to function and attempted to fulfill the tasks assigned to them by politicians. They implemented the frequently revised regulations and the repeatedly amended Infection Protection Act, including the so-called “Federal Emergency-Brake 2021” [22] and further “mini-lockdowns”, school closures, testing requirements, mask mandates, and even facility-specific vaccination requirements in 2021, imposing restrictions on fundamental rights. They further restricted freedoms on the basis of legal regulations through isolation and quarantine orders; also healthy children (as contact persons) as well as children who tested positive with questionable rapid tests were excluded from attending school and visiting all sports and leisure facilities. Elderly people living in care facilities were isolated in their rooms for long periods without any possibility of receiving visitors. In the nursing and medical sectors, healthy, asymptomatic employees were also quarantined as contact persons and unvaccinated staff were excluded, which at least partially jeopardised the care of patients and those in need of care. The health authorities worked at least in part beyond their own limits in order to fulfil the requirements imposed by politicians, in some cases above and beyond the call of duty.

During the COVID-19 pandemic, the reporting data of those who tested positive was used to inform the public and guide government measures, rather than the surveillance data implemented after the 2009 influenza pandemic (see above), even though this data (see Table 1 (Tab. 1)) continued to be collected and published weekly by the RKI [23]. This data would have allowed a good comparison with previous years, as it had been collected using identical methods for years, thus avoiding bias in this data due to changed collection methods (e.g., changed testing requirements) [24]. 

Based on the notifications and contact tracing, health authorities were able to recognize early on that schools were not hotspots. Infections in schools were rare compared to infections in private or family settings [25], [26], [27], [28], [29], [30], [31], and the costs and collateral damage (especially the stigmatization of children) of mandatory mass antigen-testing in schools were disproportionate to its benefits [30]. The mandatory notifications quickly showed that vaccination did not achieve sterile immunity, as second and multiple infections and transmissions occurred even after vaccination [32], [33]. Mandatory vaccination was therefore not justifiable from a scientific or legal standpoint. In their discussions with parents as part of contact tracing, health authorities learned about the problems children and parents were experiencing with school closures, and the other option learning one day in school, the other day home. Through their contacts with elderly care facilities, the PHAs were aware of the challenges faced by home managers in ensuring the care of residents in the face of staff quarantine, they knew of the impossibility of meeting the psychosocial needs of residents, and learned of the consequences of isolating elderly people, which all too often robbed them of their courage and will to live and deprived them of their dignity. The health authorities were therefore aware of the “side effects” of their measures earlier than others [34], [35]. Such data and findings from the health authorities were rarely published in a timely manner, and in most cases not at all (exceptions were, for example [25], [26], [27], [28], [29], [30], [31], [32], [33], [36], [37], [38]). 

In some cases, the questionable nature of such measures, which deviated from the evidence in the existing pandemic plan of the health authorities, was recognized and criticized by PHA experts. Within the authorities, political superiors were asked to introduce changes. This critique was not made public, and if it was, there was a risk of being dismissed or transferred [39], [40]. An exploratory survey among PHA employees showed that more than 60 medical colleagues left (or were forced to leave) the PHAs during the pandemic, with political conflicts being a frequently cited reason [41].

The German Association of Public Health Service Physicians (BVÖGD) successfully campaigned for better recognition and funding for public health departments. Back in the summer of 2020, a €4 billion Pact for the Public Health Service [42] was passed, which has since led to significantly better financial and personnel resources for public health departments [43]. However, the PHAs did not receive any expert support from the BVÖGD. Contrary to expert knowledge, in January 2021 the BVÖGD advocated for a further, more stringent lockdown in order to achieve a notification incidence of less than ten new infections per 100,000 inhabitants per seven days [44]. In the fall of 2021, the BVÖGD advocated for the prevention of every single infection – which is impossible – instead of referring to the pandemic preparedness plan, and called for institution-based mandatory vaccination [45]. In the fall of 2022, it called for the mandatory wearing of FFP-2 masks on public transportation [46]. Such demands were not scientific- or evidence-based, did not correspond to the pandemic preparedness plan, and also contradicted the published statements of scientific societies such as the German Society for Hospital Hygiene (DGKH) and the German Society for Pediatric Infectious Diseases (DGPI) [47], [48], [49]. 

### Legal principle of proportionality

The objective (as a basic prerequisite for reviewing the proportionality) of the legally prescribed measures often remained unclear: Was it

- a) to prevent infections in schools (safe schools),
- b) to prevent infections in children for their own protection (protecting children’s health), or
- c) to prevent infections in children for the protection of others (prevention of serious illness in vulnerable persons [parents, grandparents]) through transmission from infected children?

School closures would be an appropriate means of achieving objective a). However, since hygiene measures were proven to effectively prevent transmission in schools, school closures were not necessary. School closures would not be an appropriate means of achieving objectives b) and c), as they would only prevent the (few) infections in school buildings, but not infections among children in general, since children usually became infected outside of school during their leisure time. Therefore, they were also not necessary. 

Furthermore, the appropriateness of all objectives should have been questioned, since children infected with SARS-CoV-2 generally either do not fall ill or only become mildly ill. On the other hand, the undesirable effects of school closures and restrictions on children’s education, psychosocial development, and mental and physical health can be considerable. In any case, the question as to the appropriateness of (prolonged) school closures would have had to be answered with „no“ as well.

Furthermore, question c) involves a massive restriction of children for the benefit of others, which is incompatible with the right to the best interests of the child.

Children were still being quarantined as contact persons in schools by many health authorities, even though milder measures [29], [30] had proven to be similarly effective in preventing infections in schools and despite the fact that studies had already shown the significant side effects of these measures at an early stage [50]. In 2022, health authorities continued to exclude children from school (until retesting with the PCR test) based on the results of mandatory rapid testing in schools, although they must have known that these rapid tests were very unreliable, invalid, and not approved, as they produce very low positive predictive values, especially at low prevalence [30], [31].

Further examples include

- Isolation of residents who tested positive and quarantine of contact persons in nursing homes. The resident’s freedom of movement was often restricted for weeks, although the significant adverse effects on their health and well-being had been reported and warned against early on [35], [51].
- Implementation of facility-related vaccination requirements: Health authorities imposed fines and even bans on working for staff in medical and nursing facilities who were unwilling to be vaccinated. However, since vaccination does not result in sterile immunity, the vaccination requirement was not a necessary means of preventing transmission to patients and those in need of care, unlike the “milder measure” of requiring staff to wear mouth-nose protection correctly.
- Implementation of the recovery certificate shortened to 3 months. From a scientific point of view, this was more than questionable with regard to good immunity after a natural infection, and from an immunological point of view it was counterproductive, as the booster vaccination was mandatory after three months and this can even impair the development of good long-term immunity [52].

During the pandemic, laws and regulations were enacted and modified in a very short time. There was no evidence that PHAs had carried out assessments of proportionality and observed the prohibition of excessive measures, i.e., the obligation to use the mildest means possible. In some cases, the requirements were exceeded. For example, shortly before Christmas 2022, a head of a PHA imposed a fine for non-compliance with the institution-related obligatory vaccination, even though this law was known to be scheduled to expire eight days later. In another PHA, the mask requirement was still in place in February 2023, even though it had not been required in businesses, shops, etc. for a long time. One PHA was still planning a comprehensive four-stage scenario for the winter of 2022/2023, requiring between 200 and 500 full-time staff to combat the pandemic [53]. 

On the other hand, there are known cases where health authorities protested. As early as spring 2021, for example, some authorities suspended general contact tracing, thereby implementing the pandemic plan in this respect. Since older people were much less likely to become seriously ill with the Omicron variant, in 2022, their isolation in nursing homes was sometimes no longer restricted to their own rooms but to the entire living area, which significantly improved their freedom of movement and quality of life [54]. In some cases, response deadlines were set for the implementation of facility-based vaccination requirements until the expiration of the regulation was foreseeable. However, such approaches were not openly discussed – with a few exceptions, such as a position paper issued by the Baden-Württemberg PHA in March 2022 [55].

### Lessons from history

During the pandemic, PHAs sent letters such as the following to the family of a five-year-old child who had been in contact with a person who tested positive. “As the parent of a close contact, you do not have to quarantine. However, make sure that your child isolates himself from you as much as possible. [...] Please follow all the rules so that you do not infect anyone. If you violate the rules of the general directive, the fine can be up to 25,000 Euros (§73 (1a) No. 6 IfSG). [...].” The question was raised: ”What kind of letters would PHA employees send if they were asked to do so? Would there be a limit, and where would it be?” [56].

Without explicitly mentioning it, this question alludes to the history of health authorities under Nazi rule. This has been comprehensively researched in recent years on behalf of the BVÖGD [57], also with a view to learning lessons for the future. To this end, the BVÖGD has awarded the Johann Peter Frank Medal twice: in 2018 to Dr. Johannes Donhauser and in 2025 to Prof. Sabine Schleiermacher.

In his acceptance address, “The inviolability of individual civil liberties; lessons for the PHA from its Nazi past,” [58], Donhauser wrote: “Those of us currently working in the public health service (PHS) should always act in the knowledge that we are a responsible part of an administration that, as a bureaucracy, is subject to the principle of legality ... We are all familiar with the principles, such as the prohibition of excess (*Übermaßverbot*) and acting according to dutiful judgment, taking into account the individual case.” While Donhauser had pointed to the inviolability of individual freedoms and the prohibition of excessive measures as lessons for the PHS when reviewing the activities of the health authorities [58], individual freedoms were very quickly restricted during the pandemic without any apparent consideration of proportionality. Only a few health authorities raised internal objections. The BVÖGD welcomed the restrictions on freedom and in some cases called for even tighter measures [44], [45], [59]. Against this backdrop, Donhauser critically noted recently: “The PHS allowed itself to be willingly instrumentalised during this pandemic because, particularly in this exceptional situation, the public and, above all, political pressure was enormous” [60].

In her acceptance address, Schleiermacher showed how “uniform, bureaucratic, formalized administrative action, ... (suggested) neutrality and correctness” and she continued: “However, while focusing on this activity, it is possible to lose sight of its goal and effects on the people affected, and a self-referential momentum can develop.” She was concerned with “... how and how quickly ethical values and norms are changed and shifted depending on context. However, these are not abrupt incidents or inevitable events, but developments based on decisions. Critical engagement with history can, where appropriate, help to identify patterns and structures, make these discoveries available as questions for the present, and thus enable informed decisions” [61].

## What could be the underlying causes?

Why was so little attention paid by the PHAs to expertise, legal knowledge and principles – as well as historical experience – during the COVID-19 pandemic?

Mass phenomena, which Canetti described as early as 1980 in his book “Crowds and Power” [62], may offer an explanation. According to his theory, simplified messages and threat scenarios can lead to a loss of critical thinking and a tendency to follow the crowd. Threat scenarios can trigger uncertainty and fear, blurring individual barriers in favor of collective reactions and causing individual actions to follow a collective dynamic in which restraints and differentiating boundaries disappear. During the pandemic, fear was created in many countries through a threat scenario, and appeals were made to collectivism, obedience, and solidarity. Hence, mass psychology and group processes took hold of societies – a “collective spirit” emerged, while independent thinking, personal responsibility, and social norms were lost. This could explain the de-individualization and aggression observed toward those who were thinking differently, e.g., in dealing with people who refused vaccination and the unvaccinated, a “group made into scapegoats” [63].

In Germany, too, a threat scenario had spread throughout almost the entire society within a very short time in the spring of 2020. Under the “corona narrative” [64], combating the virus became the one and only goal, which (supposedly) required maximum measures and restrictions on freedom, without considering the consequences for society as a whole. 

In the first weeks of the pandemic, attention was still drawn to the pandemic plan, the risks of modelling were identified, and it was emphasized that all measures must be proportionate, appropriate, and carefully considered, as they could also have negative consequences for patient care [65]. 

When images from Italy (Bergamo) emerged in mid-March 2020, they created a threat scenario that had never existed before. The media flooded their audiences with images of critically ill patients in intensive care units, and the focus on individual fates distorted the overall picture. Most media saw themselves as the frontline in the fight against the pandemic. Politicians reacted, and a self-reinforcing feedback loop was triggered. 

By the end of March 2020, the established pandemic preparedness plan was no longer the basis for action, and a mono perspective, a tunnel view of the virus, emerged. Differentiated expert opinions (e.g., [47], [48], [49], [51], [66]) were rarely or never reported in the media, and other perspectives were mostly defamed. Citizens who asked questions and protested were labeled as contrarians and coronavirus deniers [67]. Those who did not willingly follow this policy and did not get vaccinated were discriminated against and called for to be excluded from society – by politicians of all parties, by journalists, but also by physicians and the German Ethics Council [68]. Human dignity and fundamental rights such as self-determination and freedom of opinion (Meinungsfreiheit) were ignored or subordinated to the fight against the virus by politicians, society, science, the legal system, and unfortunately by the public health services as well. 

## Looking abroad: the Swedish experience

This threat scenario, the “corona narrative” with its accompanying comprehensive measures restricting freedom, took hold in many countries around the world [64] – but not in Sweden. In Sweden, the pandemic plan was implemented, evidence- and data-based recommendations were made and communicated in such a way that the population voluntarily followed them [69], [70], [71], [72]. Large events were restricted early on, and people were advised to work from home whenever possible and to reduce their contacts. Childcare facilities and schools up to grade 9 remained open. Symptomatic cases were counted and no so-called incidences were used, which were based solely on people who tested positive (even if they were asymptomatic), although these figures were not comparable due to differences in test availability and testing strategies. There were no mandatory testing or mask requirements. Despite high mortality rates, particularly among nursing home residents at the beginning of the pandemic, Sweden had one of the lowest excess mortality rates in Europe. The educational level and general vaccination rates of Swedish schoolchildren did not decline [73]. In an international comparison, Sweden had the highest “negative excess mortality” in Europe (–3.5%) by mid-2023, slightly surpassed only by New Zealand (–3.6%), but significantly lower than, for example, Germany (+2.8%) [74]. Life expectancy among the Swedish population increased by 0.11 years from 83.26 years in 2019 to 83.37 years in 2021. In contrast, 20 European countries saw an average decrease of 0.18 years – in Germany, which had a comparatively low life expectancy of 81.35 years in 2019, life expectancy decreased by 0.20 years between 2019 and 2021 [75]. 

The structures for managing a pandemic in Sweden are very different from those in Germany. The central player in Sweden is the politically independent national health authority (Public Health Agency of Sweden). “The health authority is independent from the government. [...] The Government has no power to intervene in an agency’s decisions on specific matters relating to the application of the law or the decisions within its authority. Collective Government decision-making and the ban on instructing agencies on individual matters illustrate the prohibition of ‘ministerial rule’, as it is often called” [71]. 

Within this mandate, the Public Health Agency of Sweden has acted in close contact with the regions, municipalities, and other government agencies, but also with many private actors. All regulations were forwarded to the interest groups affected by the regulation for comment before a final decision was made [71] – see also [76]. Individual municipalities were able to order additional measures depending on the local/regional situation. This made the above-mentioned successes possible.

## What lessons can be drawn for the future of public health authorities in Germany?

Currently, German PHAs vary greatly in terms of staffing, ranging from a few to several hundred employees. Not in all federal countries is the expertise of a physician specialist in medical public health care required to be the head of a PHA, and even where this is mandatory, trained and experienced specialists are sometimes not available. After the pandemic, calls were made for better resources for PHAs (finances, personnel, digitalization, scientific expertise), better crisis management, better coordinated communication channels, the establishment of an action-oriented scientific system in and around the PHA and a consistently multi-professional PHA [77], [78]. However, the question arises as to whether the PHA would be able to act more professionally and legally under these conditions, as the pandemic showed that scientific rigour and evidence were often not taken into account, not only in the PHAs and RKI [79], [80], but also in general [81].

In Germany, PHAs are subordinate to all levels of government, regardless of whether they are municipal or state health authorities. The expertise available in the PHAs was often ignored by politicians during the pandemic [41], [79], [80]. For this reason, there have been calls for both the upper health authority and the local health authorities to be given professional autonomy and independence from government directives [40], [82], [83]. In order to limit the possibility of political influence in the local health authorities, it has been proposed that they be converted into public law corporations [40], a proposal that generated a broad discussion [60], [84], [85], [86], [87]. Again, the question arises as to whether the PHA would have been able to act more professionally and legally under these conditions, as this stuctural situation may be a necessary however not a sufficient condition.

The German PHAs were provided with the revised and updated pandemic plan [21]. They must also be capable to implement and use the existing and emerging evidence however. This requires not only appropriate structural conditions (see the example of Sweden) but necessarily also experienced professionals not only with broad and extensive expertise but also with personal strengths and ethical commitment to take on this task as “guardians of public health“. 

The necessary critical and constructive evaluation of the role and function of the PAHs in the COVID-19 pandemic in Germany is not only a matter of clarifying the effectiveness of individual measures from a scientific point of view, which has been done in many reviews and Cochrane reviews. It is also, and in particular, the matter of addressing the broader societal question of how, even in times of pandemic, evidence and experience rather than threat scenarios can remain the basis for decision-making in PHAs – as well as in society as a whole.

We suggest that the PHA take the initiative now, on its own behalf and of its own accord. Hopefully our paper can serve as a basis for a broad discussion such as that on the structural proposal above [40], either at the next BVÖGD congress or – better still – at a separate PHA conference organized for example by the recently founded scientific societies German Society for Public Health (DGÖG) and German Society for Public Health and Population Medicine (DGÖGB). In order to broaden the perspective, other stakeholders, e.g., from the Swedish Public Health Agency, should also be invited. 

## Conclusion

The analysis of the work of the PHA during the pandemic shows that the health authorities imposed extensive restrictions on freedom due to political and administrative law requirements without implementing the pandemic plan, the technical evidence and the principles of administrative law. The lessons of history were also ignored. This can be explained by mass phenomena, especially a collective threat scenario that had gripped the whole of society - including the PHA. 

After the pandemic, there was a broad discussion about improving the staffing, financial, and digital resources of PHAs, as well as about structural changes to achieve greater independence from political directives. In view of the problems presented here, this discussion should be expanded to include the question of how to ensure that PHA can implement its professional legal and ethical foundations to fulfill their role as “guardians of public health.” 

The PHA should conduct this discussion and evaluation of their role in the pandemic – on behalf of itself, society, and, in particular, the people whose freedoms have been disproportionately restricted. 

## Notes

### Authors’ ORCIDs

- Heudorf U: https://orcid.org/0000-0002-0050-8272
- Gottschalk R: https://orcid.org/0000-0003-0422-6456

### Funding

None. 

### Acknowledgments

We would like to thank Dr. Johannes Donhauser and Prof. Dr. Martin Exner for their important support, critical review, and constructive advice.

### Competing interests

The authors declare that they have no competing interests.

## Figures and Tables

**Table 1:**
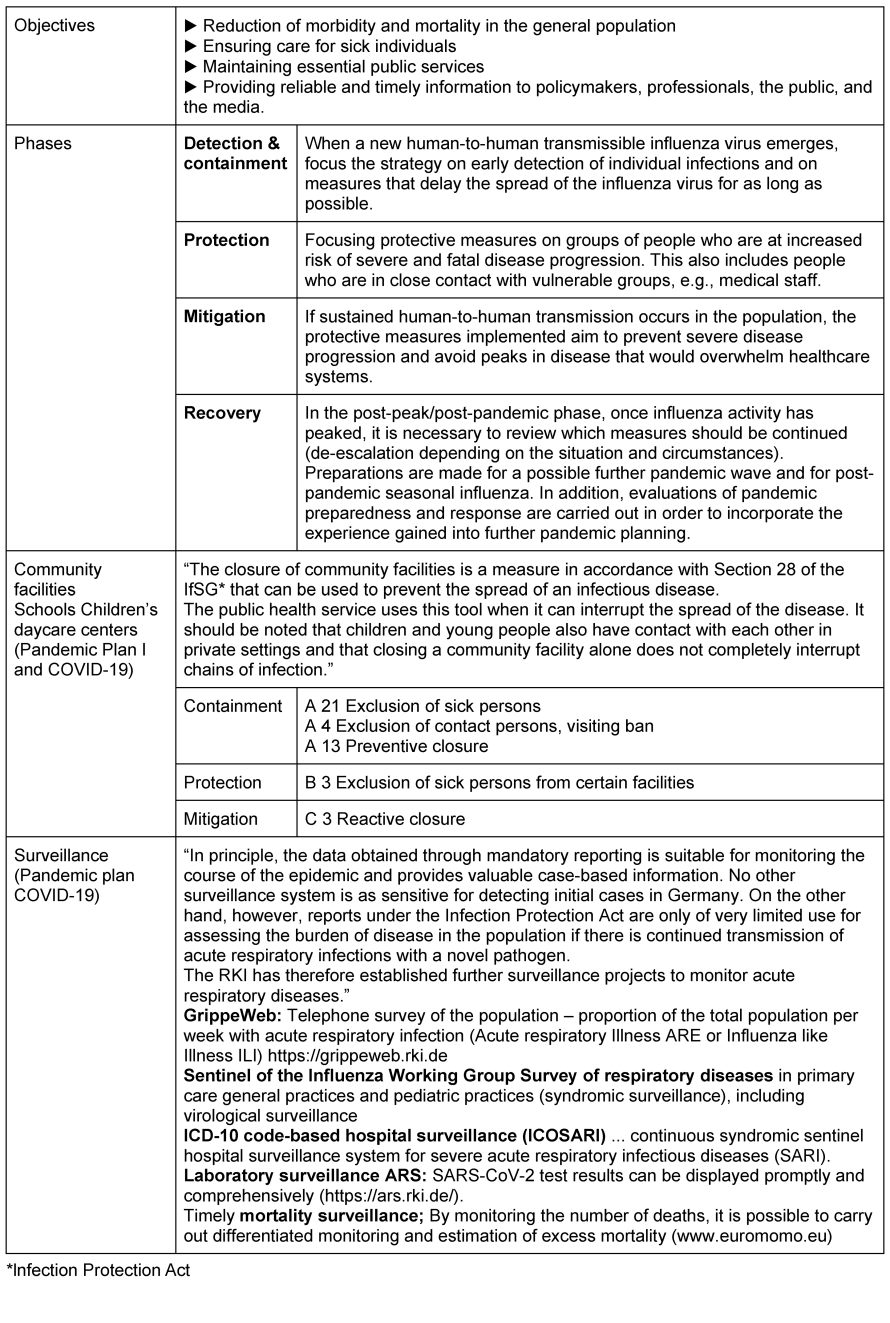
COVID-19 pandemic preparedness plan: objectives, phases, specific measures, and surveillance [16], [21] (excerpt)
